# Danish Medical Institutions

**Published:** 1903-10-03

**Authors:** 


### DANISH MEDICAL INSTITUTIONS.

### I.—THE FINSEN INSTITUTE.

When Charles II. married Catherine of Braganza it became the fashion in English society to study Portuguese. The English did not follow this courteous fashion when our present King married Princess Alexandra of Denmark, but as, on the other hand, the Danes set themselves energetically and almost universally to the study of English, it is possible to gain a knowledge of Danish life and institutions with little or no knowledge of the language, if only one has an introduction to some of the people who are interested in the subjects one wishes to study. I formed one of a party organised by Miss Butlin, an English lady who has lived much in Denmark and knows many prominent Danes, and through this I was easily enabled to gratify my desire to become acquainted with the medical charities of Denmark, especially those which differ from our own.

My first visit, however, was paid to an institution from which our own similar one cannot differ greatly, inasmuch as it was copied from the Danish one—the Finsen Institute for the cure of lupus. The environment, however, differs greatly. Instead of the noise and dust of the Whitechapel Road, there is the peace of the Rosenvanget Allée, and instead of being an annexe to a large institution, the original Finsen Institute stands alone in a garden, and surrounded by the gardens of the neighbouring villas, where, when I visited it, the dahlias and gladioli were in full bloom, and the rich scarlet berries of the mountain ash were tempting the birds to come and sing the "tak for maal"—thanks for food—which every Dane, be he guest or member of the family, says at the end of an entertainment. From the Sound, not far off, sweeps the fresh clean air, rich in ozone, and when, after their hour of treatment, the patients rest in the long verandah, lounging in deck chairs and sometimes drinking milk; they are thus placed in conditions which are exceptionally favourable for maintaining the good effects of treatment and strengthening the constitution against the disease. To this I attribute the fact that none of the cases which I saw in Copenhagen, nor any of those whose portraits are kept in the Institute, seemed as bad as those which I have seen in the London Hospital. This opinion was confirmed by the statement of an English lady who had been treated at the London Hospital, but who had now come to Copenhagen and found that she was making much greater progress in Denmark than in England.

In the Finsen Institute at Copenhagen, 34 patients can be treated at one time. There are, in the chief ward, four of the large four-light lamps, and another of these is in a smaller ward, while a third contains two single-light lamps. These single lights are specially adapted to the needs of private practitioners who may wish to employ the light cure, as in most cases they will not want to treat more than one patient at a time, and these lamps naturally consume less electricity than the larger ones. All the lamps used are of Dr. Finsen's original design; the French lamp did not seem to be known to the very courteous and capable nurse who showed me over the Institute. Since with the latter it depends on the patient's own efforts instead of the nurse's will to produce the requisite local anæmia by pressing against the glass disc through which the light is concentrated, it is possible that the French light may not always prove satisfactory. On the other hand, the shortness of the *séance*—20 minutes as against an hour—is a great advantage, and the great variety of size and convexity which can be arranged for the lenses of the French light give it a great advantage for treating such parts as the corners by the nostrils and the curves around the eyes. In the Western Infirmary in Glasgow, where the French light alone is used, I have seen admirable results, but undoubtedly a light department should possess lights of both kinds, as is the case at the London Hospital.

Many of the patients receive the treatment gratis, but there are many paying patients, who are charged 100 kroner —equal to about £5 10s.—per calendar month, as against 2 guineas a week charged in London. There were several English patients at the time of my visit, and one young English lady was training as a nurse. As in London, the patients live outside the hospital. Indeed, it would not be possible to house there the 156 patients who are treated daily, but Miss Forman, the head nurse, thinks that the result of the treatment would be even more satisfactory if the patients could be kept under medical control during the whole of their stay, for by their diet and their general mode of life they can either hinder or facilitate their cure.

Although the Finsen treatment is specially associated in our minds with lupus, that is not the only disease treated in Copenhagen by the light cure. Besides lupus vulgaris and lupus erythematosus, it is used for epithelial cancer, alopecia areata, nævus, and acne.

*To be continued.*

